# PTCDA adsorption on CaF_2_ thin films

**DOI:** 10.3762/bjnano.11.144

**Published:** 2020-10-26

**Authors:** Philipp Rahe

**Affiliations:** 1 Fachbereich Physik, Universität Osnabrück, Barbarastrasse 7, 49076 Osnabrück, Germany

**Keywords:** calcium difluoride, decoupling, insulating thin film, 3,4,9,10-perylene tetracarboxylic dianhydride (PTCDA), scanning tunnelling microscopy

## Abstract

Thin insulating films are commonly employed for the electronic decoupling of molecules as they enable a preservation of the intrinsic molecular electronic functionality. Here, the molecular properties of 3,4,9,10-perylene tetracarboxylic dianhydride (PTCDA) adsorbed on insulating CaF_2_ thin films that were grown on Si(111) surfaces are studied. Scanning tunnelling microscopy is used to compare the properties of PTCDA molecules adsorbed on a partly CaF_1_-covered Si(111) surface with deposition on thicker CaF_2_/CaF_1_/Si(111) films. The identification of mostly single molecules on the CaF_1_/Si(111) interface layer is explained by the presence of atomic-size defects within this layer. Geometry-optimisation calculations using density functional theory reveal a geometry on CaF_2_(111) of nearly flat-lying PTCDA molecules with two oxygen atoms displaced towards calcium surface ions. This geometry is in agreement with the experimental observations.

## Introduction

The study of molecular adsorption on thin insulating films is motivated by the possibility to investigate and utilise molecular properties in their largely undisturbed state [1]. Molecule–thin film insulator interfaces are additionally of central importance in modern applications, for example as a critical component in organic thin-film transistors [2]. However, and despite the success of using thin insulating NaCl films for molecular decoupling [3], it is now understood that ultrathin layers are often not sufficient to truly insulate a molecular assembly. To name two examples, the conductivity through a NaCl bilayer still dominates the conductivity along a molecular wire [4] and only the usage of thick NaCl films has enabled charge stability of single molecules [5].

A particularly well-studied case of surface-specific molecular properties is the adsorption of 3,4,9,10-perylene tetracarboxylic dianhydride (PTCDA) on metal [6–12], semiconductor [13], and insulator surfaces [14–19], as well as the deposition on conducting surfaces covered by insulating thin films [20–24] or two-dimensional materials [25]. It is also noteworthy that PTCDA on bulk NaCl(001) surfaces shows long-range order with a dewetting transition at a certain coverage [18], while experiments using NaCl thin films on Cu(111) revealed an absence of long-range order with molecules rather bound to step edges [22]. Explanations for this different behaviour include the slight difference in the lattice constants of bulk and thin-film NaCl and the extension of the metallic state across the thin film. A difference in the molecular adsorption properties on insulating thin films of varying thickness has also been found for PTCDA molecules on partially KBr-covered Ag(111) surfaces [24] as well as for cyanoporphyrin molecules on KBr-covered Cu(111) surfaces [26].

Here, the understanding of molecular adsorption on insulating thin films is extended by studying an insulator-on-semiconductor system, namely CaF_2_ thin films grown on Si(111) surfaces, with PTCDA as the probe molecule. In particular, the adsorption properties of PTCDA on CaF_2_/CaF_1_/Si(111), CaF_1_/Si(111), as well as Si(111)-(7 × 7) surfaces are experimentally investigated by high-resolution scanning tunnelling microscopy (STM) and the adsorption geometry on a CaF_2_(111) slab is theoretically modelled using density functional theory (DFT). A prominent difference of the molecular properties on the different surface areas is the presence of mostly single molecules in CaF_1_/Si(111) regions, while ultrasmall molecular assemblies are experimentally observed on thicker CaF_2_ films. A rather flat-lying geometry is found from geometry-optimisation calculations of a single PTCDA molecule on a CaF_2_ slab using DFT, whereby an interaction between two carbonyl oxygen atoms and two surface calcium ions leads to a slight deformation of the PTCDA molecule.

## Methods

Sample preparation and STM experiments were performed under ultrahigh-vacuum conditions. Highly B-doped p-type Si(111) samples (Institute of Electronic Materials Technology, Warsaw, Poland) were used as substrates. The (7 × 7) reconstruction was formed by flash cycles and the (7 × 7) surface quality was checked by STM imaging.

CaF_2_ material (cleanliness 99.9%, Alfa Aesar, Kandel, Germany) was deposited from an e-beam evaporator (type EFM3T, Focus GmbH, Huenstetten, Germany) on silicon samples held at about 600 °C by direct-current heating. Further details on the sample preparation and CaF_2_/Si(111) surface properties can be found in [27–29]. PTCDA molecules were deposited from custom-built Knudsen cells heated to 290–300 °C. Samples were held at room temperature during deposition unless noted otherwise. STM data were acquired at 77 or 5 K using a ScientaOmicron qPlus LT AFM/STM operated by a MATRIX controller and an atom-tracking system [30]. Image data were acquired in constant-current or constant-height mode while applying a sample bias *U*_b_. Gwyddion [31] was used for the image data analysis.

Density functional theory calculations were performed using cp2k (http://www.cp2k.org) [32] and parameters similar to previous calculations were chosen [33]. The MOLOPT short-range basis sets of double-ζ quality [34–35], mixed Gaussian and plane waves, GTH potentials [36], the PBE GGA functional [37], the Γ point sampling, the Grimme DFT-D3 dispersion correction [38], and a basis-set superposition-error corrected calculation of the adsorption energies using the counterpoise method [39] were used. Geometry-optimisation calculations were performed for a single PTCDA molecule adsorbed on a (6 × 6) slab with a thickness of three CaF_2_ triple layers. The lowest triple layer was held fixed. A tolerance of 10^−4^ Ha/Bohr was used.

## Results and Discussion

**Figure 1:**
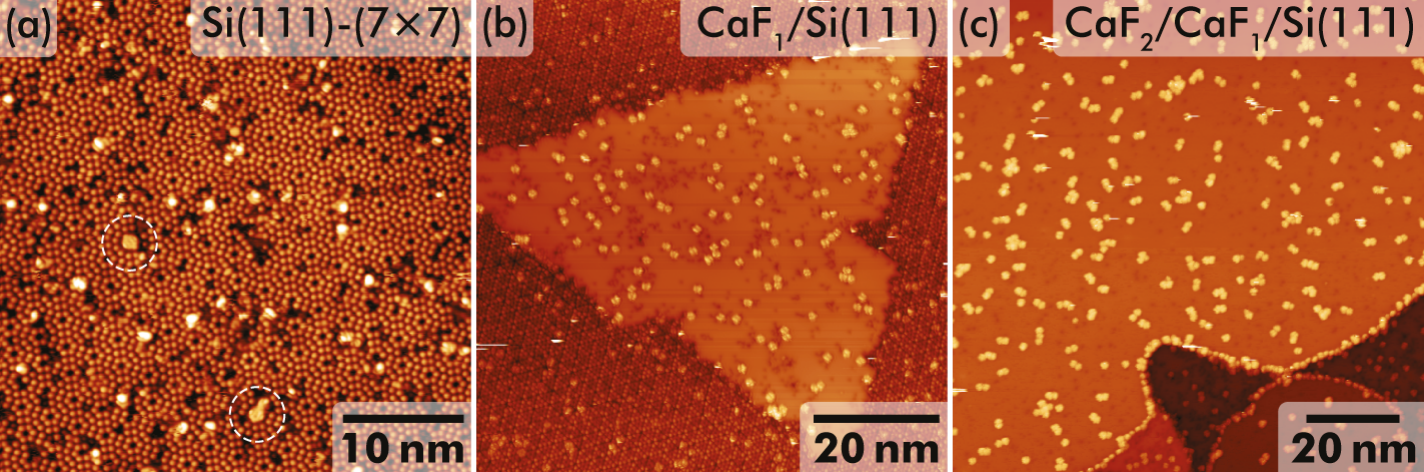
STM images of PTCDA molecules on (a) Si(111)-(7 × 7), (b) partly CaF_1_-covered Si(111), and (c) CaF_2_/CaF_1_/Si(111) surface areas. Imaging parameters: (a) dynamic STM, *U*_b_ = 3.0 V, *I_t_* = 50 pA; (b) STM, *U*_b_ = −3.0 V, *I_t_* = 50 pA; (c) *U*_b_ = −3.0 V, *I_t_* = 50 pA.

PTCDA adsorption was studied by STM after deposition on partly CaF_1_-covered Si(111)-(7 × 7) and CaF_2_/CaF_1_/Si(111) surfaces. Representative overview images acquired at 77 K on the three different surfaces areas are reproduced in Figure 1a–c. In agreement with an earlier study [13] of PTCDA on Si(111)-(7 × 7), molecules bind in various geometries to the pristine silicon surface as apparent from Figure 1a. Among these geometries, a distinguished adsorption position on top of the (7 × 7) corner hole stands out. Two examples are marked by white ellipses in Figure 1a.

The growth of ordered CaF_2_ films on Si(111) requires the formation of a CaF_1_ interface layer as the first step. This interface layer is generated by an interface reaction between CaF_2_ and the silicon surface [28–29], where surface temperatures around 600 °C during deposition facilitate the dissociation of CaF_2_ to CaF_1_ and F. The dissociated fluorine atoms mostly desorb from the surface, likely in the form of Si*_x_*F molecules [28–29]. Thicker CaF_2_ layers can then be grown stoichiometrically on the interface layer by successive CaF_2_ deposition. The CaF_1_/Si(111) surface has a (1 × 1) termination after etching the Si(111)-(7 × 7) reconstruction.

After PTCDA deposition, individual double-lobe features are apparent in STM at negative sample bias on the CaF_1_/Si(111) areas (see also Figure 1b), in addition to the dark spots that were identified before as single atomic-size defects within the CaF_1_ interface layer [27]. Each of these double-lobe features is tentatively assigned to a single PTCDA molecule. In contrast, very small assemblies formed by a few bright protrusions are observed on CaF_2_ surface areas, see Figure 1c. In these data, a single protrusion is again assigned to an individual PTCDA molecule. Neither long-range order nor extended island formation is apparent in any surface area.

**Figure 2:**
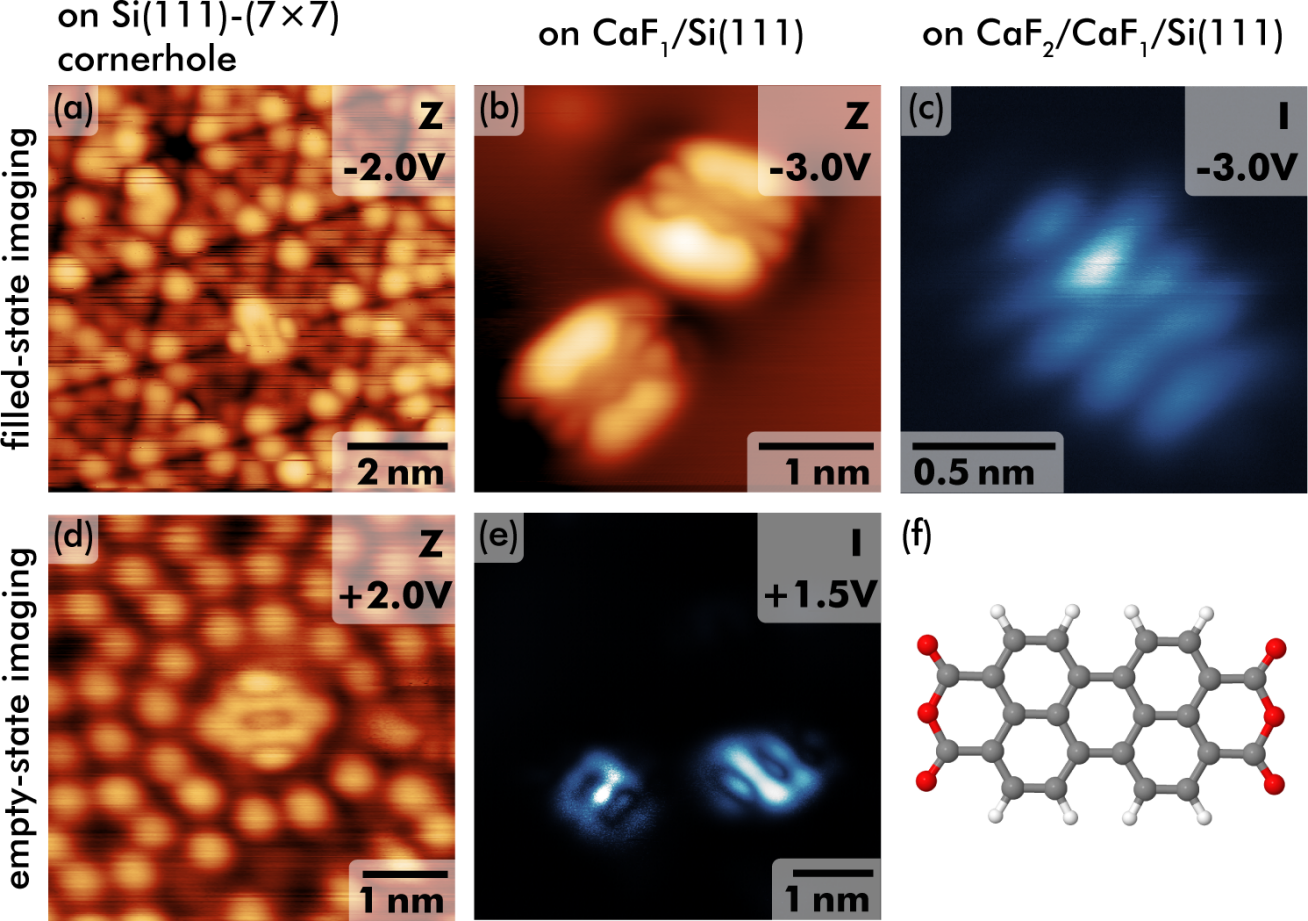
STM images of individual PTCDA molecules on different surface areas. Imaging of PTCDA on a Si(111)-(7 × 7) cornerhole position at (a) negative and (d) positive sample bias (sample bias indicated in the upper right corner). (b) Topography-mode image at negative bias and (e) constant-height mode imaging at positive bias of PTCDA on a CaF_1_/Si(111) surface area. (c) Example of imaging PTCDA on a CaF_2_/CaF_1_/Si(111) multilayer in constant-height mode. (f) Model drawing of a single PTCDA molecule (black: carbon, white: hydrogen, red: oxygen atoms).

Examples of imaging individual PTCDA molecules with STM are shown in Figure 2. The images of the cornerhole geometry on Si(111) taken at negative (Figure 2a) and positive (Figure 2d) sample bias both resemble the previously observed contrast for PTCDA at a cornerhole position at negative sample bias [13], where the molecule appears in the form of a central ring surrounded by two bright, elongated lobes. The previously observed striped structure measured at positive sample bias of +0.4 V [13] was not found as imaging was not stable at small bias in the present experiments.

On the CaF_1_ interface layer, a double-lobe feature (see also Figure 1b) is the prevalently observed shape of PTCDA, similar to the coffee bean-like shape measured by STM on KCl(001)/Ag(001) [21] or on KBr(001)/InSb(001) [23] thin films. A similar pattern was also observed in STM imaging of PTCDA/Ag(111) with an s-tip at a bias between −0.4 and −0.5 V [12] and explained by strong domination of the LUMO. In contrast, imaging at larger tip–sample distance and the according loss of sensitivity to intramolecular features was given as an explanation for the prevalently observed double-lobe feature on KCl(001)/Ag(001) [21]. The experiments are in agreement with these findings as the substructure between the two double lobes (see Figure 2b) was only apparent in very few cases; most data only reveal two bright lobes. Molecules were often manipulated and tip changes occurred when reducing the tip–sample distance for improving the STM contrast. The substructure enclosed by the double lobes is reminiscent of the LUMO charge density distribution [10], yet, at negative sample bias. Possible reasons include a non-negligible electronic coupling across the ultrathin film, coupling to defects within the CaF_1_ layer, or charge transfer into the LUMO. Imaging at a positive sample bias of +1.5 V was performed in constant-height mode as the reduced sample conductivity at positive bias impeded operation in constant-current mode. Still, conductivity through PTCDA molecules is also observed at this bias voltage with an orbital structure in Figure 2e that is different from the filled-state image in Figure 2b. One example of a constant-height STM image acquired at a negative sample bias of −3 V on the CaF_2_/CaF_1_Si(111) thick film is shown in Figure 2c and reveals a striped structure of the PTCDA molecule. However, as the molecular appearance was different depending on the molecule and tip state, it is not discussed here in further detail.

**Figure 3:**
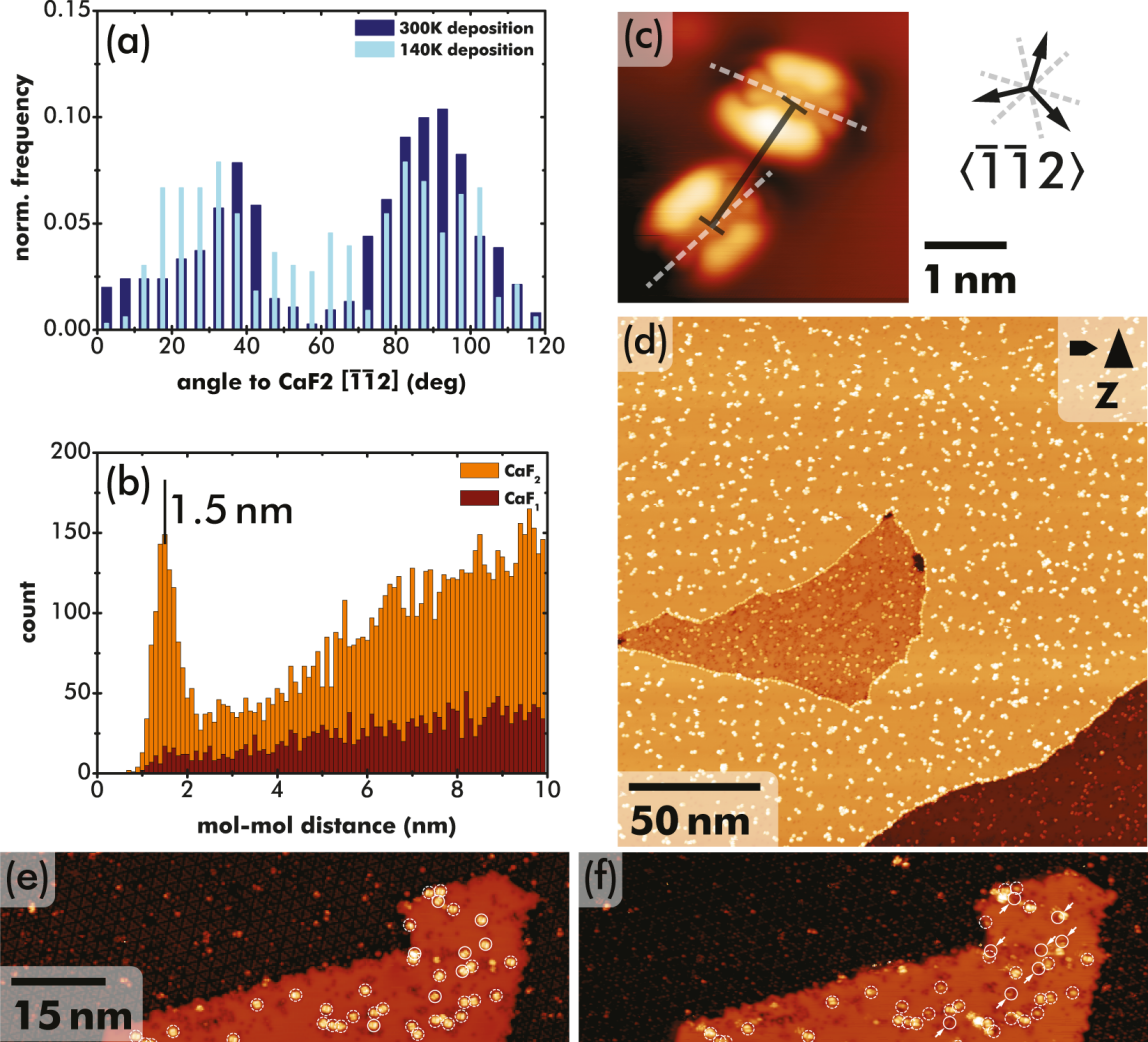
Statistical analysis of (a) the double-lobe orientation on the CaF_1_ layer and (b) the nearest-neighbour distance on CaF_1_ and CaF_2_/CaF_1_ areas. The orientation is determined by the angle between the CaF_2_ 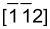 direction and the axis through the double-lobe structure, see white lines in (c). Nearest-neighbour distances are determined by the molecule–molecule distance measured from centre to centre, see black marker in (c). The topography image in (d) presenting PTCDA on a mixed CaF_2_ and CaF_1_/Si(111) surface is used for the statistical analysis in (b). STM data acquired on a CaF_1_/Si(111) surface area (e) before and (f) after accidental removal of single PTCDA molecules by the STM tip. Stationary molecules are marked by dashed white circles, removed molecules by solid circles and arrows in (f).

The STM data in Figure 1 and Figure 2 suggest preferred adsorption orientations of PTCDA in CaF_1_ and CaF_2_ areas. A detailed statistical analysis is shown in Figure 3a, where the orientation along the double-lobe shape (i.e., along the long molecular axis, see also white dashed line in Figure 3c) was measured with respect to a CaF_2_ 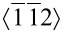 direction from more than 1000 molecules in a total of 10 images. The CaF_2_ directions were determined from filled-state imaging of the (7 × 7) reconstruction [27]. Due to the type-B epitaxy of CaF_2_ on Si(111) at the chosen growth parameters, the CaF_2_ 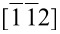 direction is identical to the Si 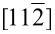 direction that was determined from STM images. Three equivalent CaF_2_ 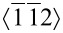 directions are depicted at the right of the exemplary image in Figure 3c. The statistical analysis was performed on two sample sets whereby the PTCDA molecules were deposited either on a sample held at room temperature (total of about 750 molecules) or with the sample support cooled by liquid nitrogen (more than 300 molecules). As apparent from the height distribution in Figure 3a, there is a strong tendency for PTCDA molecules to align under angles of about ±30° with respect to a CaF_2_ 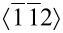 direction, especially after deposition on samples held at room temperature. The width of the peaks is attributed to uncertainties of the angle measurement and the asymmetry to a statistical artefact. However, it is also noteworthy that the intermediate minimum in the distribution is less pronounced in measurements performed after PTCDA deposition on cooled samples, which suggests an increased barrier at the estimated sample temperature of 140 K to arrive in the optimum adsorption geometry.

A second statistical analysis was performed for the nearest-neighbour distances between nearby PTCDA molecules on CaF_1_/Si(111) and CaF_2_/CaF_1_/Si(111) areas from measuring the pairwise molecular separations of a total of about 400 (1600) molecules on the CaF_1_ (CaF_2_) area in the STM image in Figure 3d. The distances were measured as the centre–centre separation, illustrated by the black line in Figure 3c. In agreement with the visual impression from the overview images in Figure 1b and Figure 1c, a preference for a centre–centre distance of about 1.5 nm is found for the CaF_2_/CaF_1_/Si(111) areas, while no clear preference is apparent from the distribution on the CaF_1_/Si(111) interface layer areas. As the surface lattice periodicities are identical for the interface and subsequent layers due to the extremely small mismatch between the silicon and CaF_2_ lattice constants [28], the absence of nearby PTCDA molecules on CaF_1_ areas can rather be explained by nucleation at defects present in the interface layer. This is substantiated by data in Figure 3e and Figure 3f, where defects in the interface layer are imaged at positions where molecules were accidentally removed in the STM scans between the two images. These positions are marked by solid white circles and arrows in Figure 3f, while stationary molecules are marked by white dashed circles in both panels. Defects are imaged in STM as black depressions although they are of atomic size [27]. In contrast, the CaF_2_ surfaces are mostly defect-free.

**Figure 4:**
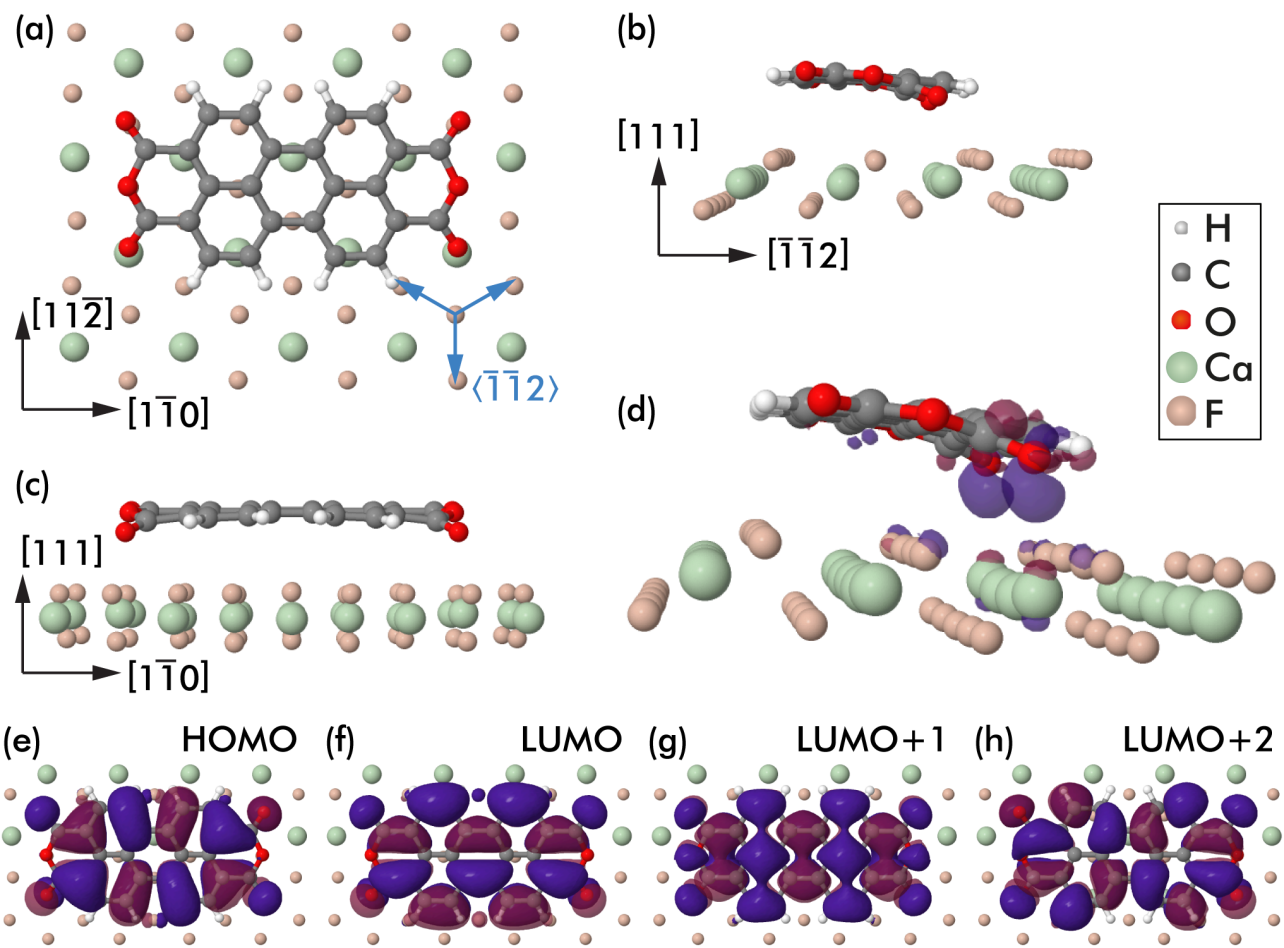
Geometry-optimised adsorption geometry of a single PTCDA molecule on a CaF_2_(111) slap in (a) top view and (b,c) side views. (d) Perspective view of the geometry with isosurfaces at −0.01 e/Å^3^ (red) and 0.01 e/Å^3^ (blue) of the electron density difference Δρ included. (e–h) Isosurfaces of the molecular orbitals HOMO, LUMO, LUMO+1 and LUMO+2 of the PTCDA/CaF_2_(111) system.

The PTCDA adsorption geometry was further investigated by using density functional theory-based calculations. Geometry-optimisation was performed for a total of seven starting geometries with flat-lying PTCDA molecules positioned under different angles and at various lateral positions on the three-layered, (6 × 6) CaF_2_(111) slab (only the top layer is shown in Figure 4). Five starting geometries relaxed into the same final geometry shown in Figure 4 and two geometries were trapped in local minima of the energy landscape and are, thus, not further discussed here. The geometry in Figure 4 with a BSSE-corrected binding energy of −1.66 eV is assessed to be the optimum adsorption geometry for a single PTCDA molecule on a CaF_2_(111) surface. In this geometry, the PTCDA molecule is aligned with the long axis along the 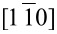 direction, which corresponds to an alignment with an angle of 30° off a CaF_2_ 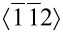 direction (directions are included for clarity in Figure 4a). This orientation is in full agreement with the experimental observation in Figure 3a, where maxima in the orientation histogram were found at angles of about 30° off the 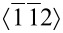 directions.

The PTCDA molecule is in a nearly flat-lying geometry, with the exception that two of the four carbonyl oxygen atoms are displaced out of the PTCDA plane towards a surface calcium ion. An attraction between the molecular oxygen and surface calcium atoms is suggested from an oxygen–calcium distance of about 2.8 Å. Note that this distance is larger than 2.4 Å recently found for the oxygen–calcium distance of a carboxylic acid moiety of a ferrocene derivative that binds vertically to CaF_2_(111) [33]. The oxygen displacement does not occur on the other side of the molecule where the carbonyl oxygen atoms are located on top of the lower-lying surface fluorine atoms. Thus, the larger O–Ca distance for PTCDA/CaF_2_(111) is likely the result of an interplay between the carbonyl oxygen–calcium attraction and the repulsion between the other oxygen atoms and the surface fluorine atoms, as well as the resulting deformation of the PTCDA molecule.

Figure 4d presents a perspective view of the optimum adsorption geometry, including isosurfaces at ±0.01 e/Å^3^ of the electron density difference Δρ = ρ_full_ − ρ_slab_ − ρ_PTCDA_. This difference was calculated as a difference between the electron densities of the full (ρ_full_), CaF_2_ slab (ρ_slab_), and PTCDA gas phase (ρ_PTCDA_) systems. The main finding is electron accumulation below the carbonyl oxygen atoms, in agreement with the attractive interaction with the surface calcium atom already identified before from the oxygen displacement.

Isosurfaces of the molecular orbital densities of the highest occupied molecular orbital (HOMO) as well as the three lowest unoccupied molecular orbitals (LUMO, LUMO+1, and LUMO+2) as calculated with cp2k for the PTCDA/CaF_2_(111) system are depicted in Figure 4e–h. The orbital shapes largely resemble earlier calculations of a flat PTCDA molecule in the gas phase [10], although the LUMO+1 and LUMO+2 states are here separated by about 0.1 eV and found in different order (in agreement with a previous study [10], a smaller LUMO+1/LUMO+2 energetic separation was calculated for the gas-phase molecule). The dominant contribution of the orbitals to the data shown in Figure 2 is deferred from the orbital shape: The filled-state image (Figure 2b) on the CaF_1_ thin film is of strong LUMO character, whereas the filled-state image (Figure 2c) on the CaF_2_/CaF_1_ area has similarities with the HOMO structure. The empty-state image on the thin film (Figure 2e) reveals a pattern that reminds of the LUMO+1 orbital shape, which supports the earlier suggestion that the molecular levels on the interface layer are shifted with respect to the gas-phase molecule.

A slightly deformed adsorption geometry including a small tilt of the molecule with respect to the surface plane is furthermore in agreement with the experimental observation of a slight asymmetry in the imaged lobe height, see for example Figure 2b where one of the lobes is imaged higher than the other. Last, the optimum adsorption geometry suggests a cause for the absence of long-range order: Extended PTCDA structures, such as the brickwall or the herringbone pattern, are not compatible with the three-fold rotational symmetry and the surface lattice dimensions. The adsorption geometry seems to especially block the common hydrogen bond motif between PTCDA molecules.

## Conclusion

Adsorption properties of PTCDA molecules on Si(111)-(7 × 7), CaF_1_/Si(111), and CaF_2_/CaF_1_/Si(111) surface areas were studied by STM at low temperatures. Single molecules were identified on the CaF_1_/Si(111) interface layer while ultrasmall molecular assemblies were found on the CaF_2_/CaF_1_/Si(111) areas. The presence of mostly single PTCDA molecules in CaF_1_ regions is rationalised by nucleation at defects present within the CaF_1_ interface layer. In contrast, the CaF_2_/CaF_1_ layer is mostly defect-free. A statistical analysis revealed a preferred molecular orientation of the long molecular axis along a CaF_2_ 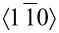 direction, in full agreement with the DFT-calculated optimum adsorption geometry. The DFT-based analysis furthermore revealed a nearly flat-lying molecular adsorption geometry with a downward displacement of two carbonyl oxygen atoms. Based on an analysis of the electron density difference, these atoms are attracted towards the surface calcium ions. A comparison of calculated molecular orbital shapes with the experimental STM data suggests a strong influence of the LUMO in filled-state STM imaging on the CaF_1_ interface layer. Instead, the absence of long-range order on the CaF_2_ films is explained by a mismatch of the common PTCDA motifs with the CaF_2_ surface structure.
